# The Impact of Endocrine Disruptors on the Female Genital Tract Microbiome: A Narrative Review

**DOI:** 10.3390/life15081177

**Published:** 2025-07-24

**Authors:** Efthalia Moustakli, Themos Grigoriadis, Anastasios Potiris, Eirini Drakaki, Athanasios Zikopoulos, Ismini Anagnostaki, Athanasios Zachariou, Ekaterini Domali, Peter Drakakis, Sofoklis Stavros

**Affiliations:** 1 Laboratory of Medical Genetics, Faculty of Medicine, School of Health Sciences, University of Ioannina, 45110 Ioannina, Greece; ef.moustakli@uoi.gr; 2 First Department of Obstetrics and Gynecology, Alexandra Hospital, Medical School, National and Kapodistrian University of Athens, 11528 Athens, Greeceeirinidrak@med.uoa.gr (E.D.); kdomali@yahoo.fr (E.D.); 3 Third Department of Obstetrics and Gynecology, University General Hospital “ATTIKON”, Medical School, National and Kapodistrian University of Athens, 12462 Athens, Greece; apotiris@med.uoa.gr (A.P.); thanzik92@gmail.com (A.Z.); isanagnostaki3@gmail.com (I.A.); pdrakakis@med.uoa.gr (P.D.); 4 Department of Urology, School of Medicine, Ioannina University, 45110 Ioannina, Greece; zahariou@otenet.gr

**Keywords:** vaginal microbiota, hormone disruption, microbial dysbiosis, bisphenol A, parabens, phthalates, reproductive health, environmental exposure

## Abstract

Background/Objectives: Endocrine disruptors (EDs) are xenobiotic chemicals that disrupt hormone signaling and homeostasis within the human body. Accumulative evidence proposes that EDs could affect systemic hormone balance and local microbial communities, including the female genital tract (FGT) microbiome. The FGT microbiome, and especially the vaginal microbiota, contributes significantly to reproductive health maintenance, defense against infection, and favorable pregnancy outcomes. Disruption of the delicate microbial environment is associated with conditions like bacterial vaginosis, infertility, and preterm birth. Methods: The present narrative review summarizes the existing literature on EDs’ potential for changing the FGT microbiome. We discuss EDs like bisphenol A (BPA), phthalates, and parabens and their potential for disrupting the FGT microbiome through ED-induced hormone perturbations, immune modulation, and epithelial barrier breach, which could lead to microbial dysbiosis. Results: Preliminary evidence suggests that ED exposure–microbial composition changes relationships; however, robust human evidence for EDs’ changes on the FGT microbiome remains scarce. Conclusions: Our review addresses major research gaps and suggests future directions for investigation, such as the necessity for longitudinal and mechanistic studies that combine microbiome, exposome, and endocrine parameters. The relationship between EDs and the FGT microbiome could be critical for enhancing women’s reproductive health and for steering regulatory policies on exposure to environmental chemicals.

## 1. Introduction

Endocrine disruptors (EDs) are a broad category of exogenous substances that disrupt the body’s natural hormone production, secretion, transport, binding, action, or clearance for the preservation of homeostasis, reproduction, growth, and behavior [1,2]. Xenobiotics can activate or inhibit hormone activity, particularly estrogen, androgen, and thyroid hormone activity, and lead to physiological imbalances [3]. The industrial chemicals such as BPA, phthalates, parabens, dioxins, and select pesticides such as DDT constitute some examples among the most common EDs. Human exposure occurs through multiple routes, including ingestion of contaminated food and water, inhalation of airborne pollutants, and dermal absorption from cosmetic and household products [4]. A global public health concern, EDs are particularly problematic when it comes to reproductive health outcomes because of their pervasiveness in the environment and consumer products [5].

Concurrently, increased attention has been directed toward the human microbiome—the community of microorganisms, including bacteria, viruses, fungi, and their genes, living in specific environments within the body such as the gut or female genital tract—and its role in modulating health and disease. Women’s reproductive health is significantly influenced by the FGT microbiome, particularly the vaginal microbiome. The normal vaginal microbiome is usually dominated by the Lactobacillus species, for example, *L. crispatus*, *L. gasseri*, and *L. jensenii*, which produce lactic acid, hydrogen peroxide, and bacteriocins [6,7]. The metabolic byproducts of these microorganisms contribute to maintaining a low vaginal pH, thereby inhibiting the growth of pathogenic organisms. The balance of microorganisms is important for preventing pelvic inflammatory disease (PID), urogenital infections, sexually transmitted infections (STIs), and pregnancy complications like miscarriage and preterm birth [8,9].

The stability and composition of the vaginal microbiota have been found to be variable and dependent on numerous factors, for instance, age, sexual activity, hygienic practices, antibiotic usage, and contraceptive measures [10,11]. It is noteworthy that estrogen seems to play a key role in maintaining the vaginal environment. In addition to maintaining an acidic pH that favors *Lactobacillus* dominance, estrogen promotes the proliferation of vaginal epithelial cells and glycogen accumulation, which commensal bacteria metabolize to produce lactic acid and other metabolites [12]. Importantly, growing evidence supports a bidirectional relationship, where the vaginal microbiota also modulates estrogen metabolism and availability through enzymatic activity (e.g., β-glucuronidase), contributing to the so-called hormone–microbiota axis [13]. Therefore, any external substances that alter estrogen levels, such as endocrine disruptors (EDs), may disrupt this delicate host–microbiota feedback loop.

According to preliminary data, EDs may affect the FGT microbiota through a variety of mechanisms, including the direct modification of hormone receptors, changes in immunological function, and the disintegration of epithelial barriers [14]. While existing studies exploring the connection remain underdeveloped, initial research proposes that chronic or high-dose ED exposure can be linked with microbial dysbiosis and heightened susceptibility for vaginal infection, infertility, and poor pregnancy outcomes [15,16]. Despite the biological plausibility and original evidence, overall insights into the impact that EDs have on the FGT microbiome are scarce. The majority of current research addresses systemic hormone effects or general reproductive measures and seldom considers the microbiome as a mediator. In addition, differences in study design, varying exposure measurement, and scarce longitudinal data make it difficult to make definitive conclusions [17].

This narrative review aims to synthesize the current body of knowledge regarding the potential effects of endocrine disruptors on the female genital tract microbiome. We initiate our review with a discussion on the principal classes and modes of action of EDs, followed by a description of the composition and function of the vaginal microbiota. We then discuss the hypothesized modes by which EDs have the potential to disrupt the microbiome and survey the existing evidence emanating from human and animal research. This review concludes by identifying key research gaps and proposing future research directions to advance understanding of this complex and underexplored relationship. Understanding the effects of EDs on the FGT microbiome offers the potential for unraveling women’s reproductive health and informing public health policy on environmental exposures.

## 2. Overview of Endocrine Disruptors

EDs are a heterogeneous group of exogenous chemicals that interfere with the normal functioning of the endocrine system. EDs have the ability to mimic, inhibit, or disrupt hormone communications and induce imbalances in physiological processes such as growth, metabolic activity, and reproduction [2]. The endocrine system reacts extremely strongly to changes in hormone levels, and even little amounts of ED exposure can have long-lasting biological effects, especially during crucial stages like pregnancy, puberty, and fetal development [18].

### 2.1. Types and Sources of Endocrine Disruptors

EDs have diverse sources and are widespread in consumer products, industrial chemicals, agrochemicals, and air pollutants. Synthetic EDs like BPA, bisphenol S (BPS), and phthalates are widely applied in plastics, food wrap, and personal care products [19,20]. Parabens, a second group of EDs, are widespread in cosmetics, moisturizers, and shampoos as a result of their preservative action. Organophosphate and organochlorine pesticides like DDT and atrazine are also established EDs that remain persistent within the soil and water and find entry into the food chain [21]. Additionally, synthetic substances such as dioxins, flame retardants like polybrominated diphenyl ethers (PBDEs), and polychlorinated biphenyls (PCBs) have been shown to disrupt hormones [22].

The exposure to such chemicals is global and can occur through various routes. EDs can be ingested by the human being through contaminated water and food, be inhaled through suspended particulates, or be absorbed transcutaneously [23,24]. Accordingly, EDs have been found with measurable concentrations in the human biological samples like urine, blood, breast milk, amniotic fluid, and even follicular fluid, and hence provide evidence for the system-wide exposure and for the possible direct effect on reproductive tissues [25].

### 2.2. Mechanisms of Endocrine Disruption

EDs interfere with hormonal activity through multiple, often molecule-specific mechanisms. Among these, receptor-mediated activity is the most extensively characterized [26]. Numerous EDs have structural similarity to endogenous hormones and have the ability to interact with hormone receptors like estrogen receptors (ERα and ERβ), androgen receptors (AR), and thyroid hormone receptors. Notably, ERα and ERβ exhibit distinct tissue distributions and physiological roles: ERα is predominantly expressed in reproductive tissues and is associated with proliferative responses, whereas ERβ is more widely distributed (e.g., ovary, prostate, and CNS) and often counterbalances ERα-mediated proliferation with anti-proliferative or differentiating effects. Several EDs display isoform-selective activity, acting as agonists or antagonists preferentially at ERα or ERβ, which can lead to divergent biological outcomes. Moreover, some EDs exhibit cross-reactivity with other receptors, such as AR and PPARγ, further contributing to inappropriate hormonal signaling and disrupted homeostasis [26].

Beyond receptor interactions, EDs may also disrupt hormone biosynthesis and metabolism. Certain substances disrupt essential steroidogenesis-related enzymes, including cytochrome P450, aromatase, and 5α-reductase. These disruptions can alter the balance and availability of sex hormones, including estrogen, progesterone, and testosterone, thereby affecting hormone-sensitive tissues like the female genital tract [18,27].

Moreover, epigenetic changes are becoming established as a major mechanism for ED action. Some EDs have been found to influence DNA methylation and histone acetylation and non-coding RNA expression, resulting in the modified expression of genes that can be retained for generations. These epigenetic changes can affect the reproductive system’s development and function and can predispose to disease later on in life [28,29].

EDs can have immunomodulatory and inflammatory actions that can potentially further upset tissue homeostasis [30]. Through the modulation of cytokine production, immune cell signaling, and mucosal barrier function, EDs could set the stage for susceptibility to infections, chronic inflammation, and loss of tissue regulation—factors that are increasingly recognized as affecting the composition and stability of the genital tract microbiome [31].

Briefly, endocrine disruptors are ubiquitous, biologically active chemicals capable of influencing human health through multiple routes. Their ability for hormone mimicry, metabolic and epigenetic disruption, and immune homeostasis disruption renders them especially problematic for hormone-sensitive microenvironments like the female reproductive tract [2,32]. Of special interest, in view of the well-characterized hormone-dependence of the vaginal microbiome, is the capability for the above-described environmental chemicals to influence microbial ecology and produce reproductive disorders [7,33].

## 3. The Female Genital Tract Microbiome

The FGT is a fertile and active mucosal ecosystem that includes the vagina, cervix, uterus, fallopian tubes, and ovaries. Among the areas mentioned, the vaginal microbiome has been the most extensively studied due to its direct impact on conception success, maintenance of reproductive health, and defense against pathogenic microorganisms [34,35]. The vaginal microbiota’s architecture and activity are strongly related to the hormone system’s regulatory effect, specifically estrogen, which controls the immune system, epithelium renewal, and the nourishment needed by microorganisms to colonize [12,36].

### 3.1. Microbial Composition and Ecological Stability

Healthy vaginal microbiome is generally predominated by the species of *Lactobacillus*, particularly *Lactobacillus crispatus*, *L. gasseri*, *L. jensenii*, and *L. iners*. These species have a protective role primarily through the generation of lactic acid, which maintains the vaginal pH (approximately 3.5–4.5) low and creates acidic conditions that inhibit the growth of harmful entities. In addition to lactic acid, some *Lactobacillus* strains synthesize hydrogen peroxide (H_2_O_2_), bacteriocins, and biosurfactants, and therefore supplement their antimicrobial activity [10,37].

However, the composition of the vaginal microbiota varies considerably between individuals and is not static within or across different women. Based on microbial community profiling, researchers have classified the states of the vaginal microbiome into community state types (CSTs). The CSTs I, II, III, and V are mostly *Lactobacillus*-dominant, while CST IV displays a richer diversity of anaerobic bacteria, such as *Gardnerella vaginalis*, *Atopobium vaginae*, *Prevotella* spp., and *Mobiluncus* spp. [38,39]. The latter state, usually associated with bacterial vaginosis (BV), corresponds with a higher risk for negative gynecologic and obstetric outcomes. Vaginal microbial communities have been characterized into the CSTs based on the predominant bacterial taxa and ecological features (Table 1). CSTs dominated by *Lactobacillus* spp. are widely recognized as protective, while CST IV, marked by anaerobic bacterial overgrowth, is linked to dysbiosis and unfavorable clinical outcomes [7,40].

### 3.2. Functional Role in Reproductive Health

The vaginal microbiome offers a primary defense against microorganisms, controlling local immune responses and maintaining the continuity and integrity of the mucosal barrier [41]. It offers a basic protective function against STIs, such as HIV, HPV, and *Chlamydia*, and against urinary tract disease (UTI) and PID. In pregnancy, a balanced *Lactobacillus*-dominant microbiota is associated with decreased risks of premature birth, abortion, and low birth weight [42].

It should be noted that the vaginal microbiome is dynamic and changes in response to a variety of factors, including menstrual cycles, sexual activity, the use of antibiotics and contraceptives, and most significantly, hormonal changes that occur during puberty, pregnancy, menopause, and hormone therapy [43]. Estrogen increases epithelial storage of glycogen, which becomes a substrate for the metabolism of *Lactobacillus* and favors a protective spectrum of microbes. Conversely, low-estrogen states—the postmenopause or certain phases of the menstrual cycle—typically have high diversity among microbes and a shift to a less protective spectrum of bacteria [44,45,46].

### 3.3. Sensitivity to Hormonal and Environmental Influences

Because the vaginal microbiome is endocrine sensitive, it is especially susceptible to disruption by exogenous agents that mimic or interfere with endocrine signals [17]. Exogenous hormones, for example, those found in hormonal contraceptives, have been shown to alter microbial composition, often with beneficial effects [47]. However, under the heading of hormonal sensitivity, there is worry that exposure to environmental endocrine disruptors may cause the vaginal microbial ecosystem to shift from a balanced state to one that is dysbiotic [11]. Disruption of the composition of the microbes could destroy mucosal immunity, make the epithelia more receptive to infectious agents, and affect reproductive outcome, and potentially serve as a mechanistic mediator between exposure to EDs and gynecologic health risks [34,48].

The vaginal microbiome is an essential, hormone-sensitive part of the female reproductive system and a major contributor to reproductive health. Serious repercussions result from disturbances of the ecosystem caused by illness, immunological dysregulation, or hormone imbalance [7,49]. Deciphering the various connections between the environment and women’s health thus requires an understanding of how endocrine disruptors may affect the vaginal microbiome [50].

## 4. Mechanisms by Which EDs May Affect the FGT Microbiome

EDs have the potential to affect the FGT microbiome through various interconnected mechanisms [42]. These primarily involve the modulation of hormone activity, modifications of the immune system, and disruption of the epithelial barrier, and they all play a major part in the maintenance of the homeostasis of microbes and mucosal health [48].

### 4.1. Hormonal Modulation

The alteration of hormone signaling pathways, especially those mediated by estrogen, is one of the most notable ways that EDs impact the FGT microbiota [51]. Several EDs, such as BPA and phthalates, can bind ERα and ERβ and mimic or block native estrogen activity. Estrogen regulates glycogen storage within vaginal epithelial cells, which supplies a principal source of nutrients for the *Lactobacillus* species [52]. Through the alteration of estrogen receptor activity, EDs can potentially disrupt the supplies of glycogen and therefore affect the growth and numbers of protective populations of *Lactobacillus*. The resulting hormone imbalance can lead to the alteration of the composition of the microbes, which can facilitate the growth of the anaerobic overgrowth responsible for bacterial vaginosis and various other infections [53].

### 4.2. Immune System Alterations

The immune system plays a major part in shaping and modulating the FGT microbiome [54,55]. EDs have been seen to impact the immune system through the modulation of the production of cytokines, the activation of the immune cells, and inflammatory responses [56]. Mechanistically, certain EDs can bind estrogen receptors (ERα/ERβ) on immune cells, leading to dysregulated signaling cascades such as the Toll-like receptor (TLR)–NF-κB pathway and skewing the balance between pro-inflammatory (Th17) and regulatory (Treg) T cell responses [57]. This may result in chronic low-grade inflammation or impaired innate immune defenses, both of which can compromise the mucosal barrier’s capacity to regulate microbial communities. Consequently, a disrupted immune environment may facilitate colonization by pathogenic or opportunistic bacteria and reduce the clearance of harmful microbes, promoting microbial imbalance and increased susceptibility to infection [58].

### 4.3. Epithelial Barrier Disruption

The maintenance of the vaginal epithelial barrier provides a protective environment that avoids widespread entry of microbes and maintains tissue homeostasis. EDs can disturb the barrier through modifications in the tight junction proteins and shift the epithelial cellular renewal [41]. Vascular trauma also increases mucosal permeability, which opens the entry for invasive pathogenic bacteria and disturbs the balance within the microbiome. While the direct causal links between ED-induced barrier degradation and microbiota shifts remain under investigation, a compromised barrier is likely to amplify local immune responses and inflammation, perpetuating microbial community destabilization [59].

## 5. Evidence from Human and Animal Studies

Studies investigating EDs’ effects on the microbiome of the FGT are ongoing but have started delivering crucial evidence based on human epidemiological research and experimental models [60,61].

### 5.1. Epidemiological Studies Linking EDs to Microbiome Alterations

Changes in the vaginal microbiota have been linked to exposure to ED in a number of epidemiologic studies [11]. For instance, studies where urinary or serum concentrations of BPA, phthalates, or parabens have been compared with metabolic changes in the community have had correlations with decreased *Lactobacillus* predominance and increased occurrence of bacterial vaginosis-associated microbes [62]. These reports suggest the potential for ED exposure under natural conditions to result in microbial dysbiosis and increased urogenital infectivity. However, the vast majority of epidemiologic studies are cross-sectional and cannot be used to establish causality or temporal relationships between ED exposure and changes within the microbiome [63].

### 5.2. In Vivo and In Vitro Studies Examining Mechanistic Pathways

Animal models and in vitro systems have been invaluable for delineating potential pathways through which EDs affect the FGT microbiome. In animal models, ED exposure, such as BPA or phthalates, during the prenatal or adult periods has been shown to affect vaginal epithelial morphology, immune markers, and patterns of microbes [64,65]. Cell culture models also indicate that EDs can affect estrogen receptor activity and inflammatory cytokine production among the vaginal epithelial and immune cells and can be mediated and affect microbial colonization indirectly [66,67]. These mechanistic analyses provide biological plausibility for the epidemiologic associations and suggest pathways through which EDs can disturb microbial homeostasis [27].

### 5.3. Limitations of Current Research

Despite such advancements, several limitations hinder a comprehensive understanding of the impact EDs have on the FGT microbiome [61,68]. The majority of human research has small samples, single time point assessment, or limited characterization of microbiome dynamics [69]. Additionally, the influence of exposure dose and timing remains underexplored. EDs often exhibit non-monotonic dose–response relationships, where low-dose exposures may produce distinct or even more pronounced biological effects compared to high doses due to receptor-mediated mechanisms and endocrine feedback loops. Furthermore, critical developmental windows, such as prenatal life, infancy, puberty, and pregnancy, represent periods of heightened vulnerability, when both the host immune system and microbiome undergo rapid changes. Future research should prioritize temporal exposure models that consider dose, duration, and developmental stage to clarify how these factors interact to shape microbiota composition and function over time. Measurement of exposure often relies on the application of surrogate markers and not on the measurement of EDs directly within reproductive tissues [70,71]. Animal and in vitro models, while informative, can hardly reflect the complexity of human hormone and microbial interactions. Secondly, the diversity of EDs and the varying mechanism of action render generalizability challenging [72]. Prospective, multi-omics studies combining exposure data and clinical outcomes with microbial community profiling are needed to better establish underlying mechanisms and identify vulnerable populations [73,74].

## 6. Implications for Women’s Health

Disruption of the FGT microbiome by EDs poses profound reproductive and overall health consequences for women. Dysbiosis, often characterized as a reduction in protective species of Lactobacillus and an increase in harmful anaerobes, has been linked with a group of adverse health conditions, including BV, infertility, and prematurity [43,75].

Dysbiosis induced or exacerbated by EDs predisposes women to bacterial vaginosis, a common disorder defined by the overgrowth of anaerobic bacteria [76]. BV produces vaginal discharge and smell, but its principal relevance is the fact that it predisposes women to STIs, pelvic inflammatory disease, and complications during pregnancy [77].

Maintaining a healthy vaginal microbiome is essential for maintaining mucosal immunological homeostasis and protecting against ascending infections [78]. Disruptions relating to ED exposure might result in infertility via problematic implantation or amplified inflammation. Furthermore, the altered vaginal microbiota has been attributed to the development of preterm labor, pregnancy loss, and below-normal birth weight, further underscoring the broader impact on the outcome of pregnancy [79,80,81].

Due to EDs’ presence in the environment and in home products, exposure to EDs is common and frequently inevitable. Vulnerable groups like pregnant women, adolescents suffering hormone changes, and women with occupational exposure to chemicals might be particularly vulnerable to the ED impact on the microbiome [4]. These inequities have public health implications for reproductive justice and the need for regulatory policies for limiting harmful exposures [82,83].

**Figure 1:**
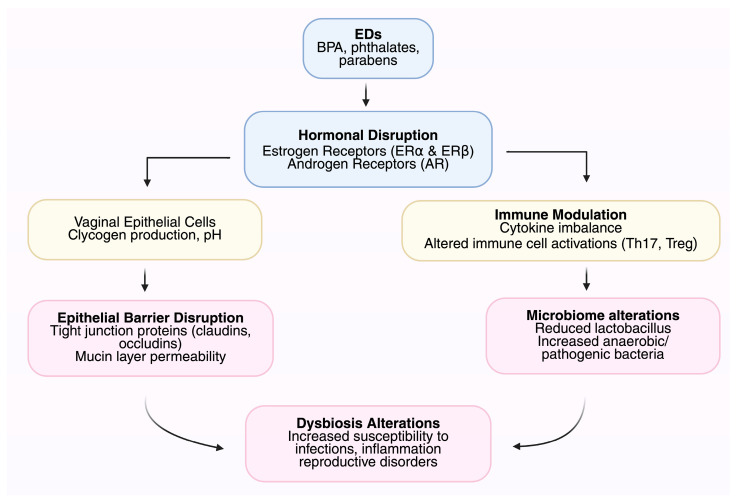
Mechanisms by which endocrine disruptors (EDs) affect the female genital tract (FGT) microbiome.

The understanding of the correlations between ED-induced changes in the microbiome and health conditions among women stresses the significance of higher awareness, preventive actions, and targeted research for mitigating risks and improving reproductive welfare across the lifespan [84]. Endocrine disruptor-induced changes in the microbiome of the female genital tract have been linked with diverse health conditions (Table 2) [85]. Bacterial vaginosis, infertility, and pregnancy disorders such as preterm birth are among them. Specific populations, such as sexually active women, pregnant women, and workers occupationally exposed, can be particularly vulnerable to the impact [86]. Figure 1 illustrates the mechanisms by which EDs affect the FGT microbiome.

## 7. Future Directions and Research Gaps

Although evidence is building up for the impact on the female genital tract microbiome of EDs, several essential gaps must be addressed for a clear picture for the understanding of their impact and for developing appropriate prevention and treatment modalities [87,88]. A pressing need centers on longitudinal studies wherein subjects are followed up for extended periods for a clearer picture of the ED exposure dynamics, microbiome changes, and the consequent health end points [89]. These have to be carried out using advanced multi-omics tools such as metagenomics, metabolomics, transcriptomics, and epigenomics, so that information on specific molecular and functional interfaces between EDs, the microbiome, and the host becomes available [90].

Another challenge is the absence of uniformity and consistency across exposure assessment methods, rendering it difficult for comparison among studies and hindering reproducibility [91]. Standardization of ED level measurement—more so for reproductive tissues and body fluids—and harmonization across methods for collecting and analyzing biomarkers will strengthen the reliability of exposure data [92]. It will also facilitate the definition of critical periods when the populations are most vulnerable to ED-induced changes in the microbiome [93,94].

Furthermore, current risk assessment tools applied today do not consider the capacity of the microbiome to adjust the toxicity of environmental chemicals. Incorporation of microbiome information into the safety profiling of chemicals could potentially advance the prediction of adverse effects and identify vulnerable populations [95]. Developing studies must discern patterns within the microbes that indicate exposure and effect, and examine microbiome-guided interventions as potential tools for the prevention of the adverse effects of Eds [96,97].

It is crucial to understand that, in addition to EDs, a variety of other factors, such as hormonal fluctuations, sexual activity, hygiene habits, antibiotic use, and the anatomical proximity of the gastrointestinal and urogenital tracts, also affect the microbiome of the female genital tract [87]. The anatomical proximity of the female rectum and vagina, for example, can enable STIs, which can change the composition of the microbiome. Disentangling the relative contributions and possible combinations of these parameters with exposure to ED should be the goal of future study [98]. Effective preventative and intervention measures would be guided by such integrated approaches, which would offer a more comprehensive understanding of the factors influencing the female genital tract microbiome [99].

Addressing such knowledge gaps will advance our understanding of the complex interplay among environmental exposures, microbial ecosystems, and women’s reproductive health and ultimately influence public health policy and clinical practice directed towards the protection of vulnerable groups.

## 8. Conclusions

Existing evidence reveals that endocrine disruptors can potentially transform the female genital tract microbiome significantly with the actions of hormone interference, immune modulation, and epithelial barrier disruption. These microbiome alterations have been attributed to the untoward reproductive health effects like bacterial vaginosis, infertility, and preterm birth, and hence present significant challenges for women’s health across the globe. The existing literature on the subject matter continues to expand, but numerous knowledge gaps persist, especially for the long-term effects, specific actions, and standardized exposure measures.

Interdisciplinary research of high caliber, which links specific exposure data with microbiome and clinical tests for the purpose of elucidating causality and informing intervention strategy, is needed. Regulatory policies must also set a process in motion for the incorporation of the microbiome as a core component for the determination of the risks deriving from endocrine disruptors. Addressing such challenges can be accomplished only through the combined efforts of scientists, clinicians, and regulators for the purpose of protecting reproductive health and countering the effects of environmental exposures on vulnerable populations.

## Figures and Tables

**Table 1:** CSTs of the vaginal microbiome. The table outlines the main CSTs based on dominant bacterial species, diversity, and pH. CSTs I–III and V are *Lactobacillus*-dominant and generally protective, while CST IV is diverse and associated with bacterial vaginosis and increased infection risk.

Community State Type (CST)	Dominant Microbial Species	Microbial Diversity	Vaginal pH	Clinical Associations
CST I	*Lactobacillus crispatus*	Low	Low (3.5–4.5)	Healthy microbiota, protective against infections
CST II	*Lactobacillus gasseri*	Low	Low	Healthy, though less stable than CST I
CST III	*Lactobacillus iners*	Low-Moderate	Slightly higher	A transitional state may coexist with dysbiosis
CST IV	*Anaerobes *(*Gardnerella*, *Prevotella*, *Atopobium*)	High	Elevated (>4.5)	Bacterial vaginosis, increased STIs, and preterm birth risk
CST V	*Lactobacillus jensenii*	Low	Low	Protective, though less common

**Table 2:** Health implications of endocrine disruptor-induced microbiome alterations in women. This table summarizes key reproductive health outcomes associated with microbiome changes caused by endocrine disruptors, highlighting the microbial shifts involved, related risks, and populations at increased vulnerability.

Health Outcome	Microbiome Change	Associated Risks	Population at Risk
Bacterial Vaginosis (BV)	Reduced *Lactobacillus*, increased anaerobes	Increased risk of STIs, pelvic inflammatory disease (PID), and symptoms like discharge and odor	Sexually active women, reproductive age
Infertility	Microbial dysbiosis, inflammation	Impaired implantation, altered mucosal immunity	Women trying to conceive
Preterm Birth and Pregnancy Complications	Shift to high-diversity, non-*Lactobacillus* communities	Preterm labor, miscarriage, low birth weight	Pregnant women
Increased Infection Risk	Disrupted microbiota and epithelial barrier	Higher susceptibility to urogenital infections and STIs	Adolescents, pregnant women, and occupational exposures
Endometriosis	Dysbiosis with increased pro-inflammatory microbes	Chronic inflammation, ectopic endometrial growth	Women with ED exposure, and reproductive age

## References

[1] Chou K. (2024). Endocrine system and endocrine disruptors. Encyclopedia of Toxicology.

[2] Diamanti-Kandarakis E., Bourguignon J.-P., Giudice L.C., Hauser R., Prins G.S., Soto A.M., Zoeller R.T., Gore A.C. (2009). Endocrine-disrupting chemicals: An Endocrine Society scientific statement. Endocr. Rev..

[3] Paramasivam A., Murugan R., Jeraud M., Dakkumadugula A., Periyasamy R., Arjunan S. (2024). Additives in Processed Foods as a Potential Source of Endocrine-Disrupting Chemicals: A Review. J. Xenobiot..

[4] Kumar M., Sarma D.K., Shubham S., Kumawat M., Vermam V., Prakash A., Tiwari R. (2020). Environmental Endocrine-Disrupting Chemical Exposure: Role in Non-Communicable Diseases. Front. Public Health.

[5] Olufemi A.C., Mji A., Mukhola M.S. (2022). Potential Health Risks of Lead Exposure from Early Life through Later Life: Implications for Public Health Education. Int. J. Environ. Res. Public Health.

[6] Papakonstantinou A., Moustakli E., Potiris A., Zikopoulos A., Tsarna E., Christodoulaki C., Tsakiridis I., Dagklis T., Panagopoulos P., Drakakis P. (2025). Behind-the-Scenes Actors in Fertility: A Comprehensive Review of the Female Reproductive Tract Microbiome and Its Clinical Relevance. Life.

[7] Chen X., Lu Y., Chen T., Li R. (2021). The Female Vaginal Microbiome in Health and Bacterial Vaginosis. Front. Cell Infect. Microbiol..

[8] Borrego-Ruiz A., Borrego J.J. (2025). Microbial Pathogens Linked to Vaginal Microbiome Dysbiosis and Therapeutic Tools for Their Treatment. Acta Microbiol. Hell..

[9] Eslami M., Naderian R., Ahmadpour A., Shushtari A., Maleki S., Mohammadian P., Amiri A., Janbazi M., Memarian M., Yousefi B. (2024). Microbiome structure in healthy and pregnant women and importance of vaginal dysbiosis in spontaneous abortion. Front. Cell Infect. Microbiol..

[10] Barrientos-Durán A., Fuentes-López A., De Salazar A., Plaza-Díaz J., García F. (2020). Reviewing the Composition of Vaginal Microbiota: Inclusion of Nutrition and Probiotic Factors in the Maintenance of Eubiosis. Nutrients.

[11] Lewis F.M.T., Bernstein K.T., Aral S.O. (2017). Vaginal Microbiome and Its Relationship to Behavior, Sexual Health, and Sexually Transmitted Diseases. Obstet. Gynecol..

[12] Amabebe E., Anumba D.O.C. (2018). The Vaginal Microenvironment: The Physiologic Role of Lactobacilli. Front. Med..

[13] Lehtoranta L., Ala-Jaakkola R., Laitila A., Maukonen J. (2022). Healthy Vaginal Microbiota and Influence of Probiotics Across the Female Life Span. Front. Microbiol..

[14] d’Enfert C., Kaune A.-K., Alaban L.-R., Chakraborty S., Cole N., Delavy M., Kosmala D., Marsaux B., Fróis-Martins R., Morelli M. (2021). The impact of the Fungus-Host-Microbiota interplay upon Candida albicans infections: Current knowledge and new perspectives. FEMS Microbiol. Rev..

[15] Bhattacharya K., Dutta S., Sengupta P., Bagchi S. (2023). Reproductive tract microbiome and therapeutics of infertility. Middle East. Fertil. Soc. J..

[16] Gullo G., Satullo M., Billone V., De Paola L., Petousis S., Kotlik Y., Margioula-Siarkou C., Perino A., Cucinella G. (2025). The Role of the Genital Tract Microbiome in Human Fertility: A Literature Review. J. Clin. Med..

[17] Plesniarski A., Siddik A.B., Su R.-C. (2021). The Microbiome as a Key Regulator of Female Genital Tract Barrier Function. Front. Cell Infect. Microbiol..

[18] Guarnotta V., Amodei R., Frasca F., Aversa A., Giordano C. (2022). Impact of Chemical Endocrine Disruptors and Hormone Modulators on the Endocrine System. Int. J. Mol. Sci..

[19] Hilz E.N., Gore A.C. (2023). Endocrine-Disrupting Chemicals: Science and Policy. Policy Insights Behav. Brain Sci..

[20] Metcalfe C.D., Bayen S., Desrosiers M., Muñoz G., Sauvé S., Yargeau V. (2022). An introduction to the sources, fate, occurrence and effects of endocrine disrupting chemicals released into the environment. Environ. Res..

[21] Ore O.T., Adeola A.O., Bayode A.A., Adedipe D.T., Nomngongo P.N. (2023). Organophosphate pesticide residues in environmental and biological matrices: Occurrence, distribution and potential remedial approaches. Environ. Chem. Ecotoxicol..

[22] Ullah S., Ahmad S., Guo X., Ullah S., Ullah S., Nabi G., Wanghe K. (2022). A review of the endocrine disrupting effects of micro and nano plastic and their associated chemicals in mammals. Front. Endocrinol..

[23] Peivasteh-Roudsari L., Barzegar-Bafrouei R., Sharifi K.A., Azimisalim S., Karami M., Abedinzadeh S., Tajdar-Oranj B., Mahdavi V., Alizadeh A.M., Sadighara P. (2023). Origin, dietary exposure, and toxicity of endocrine-disrupting food chemical contaminants: A comprehensive review. Heliyon.

[24] Ramsperger A.F.R.M., Bergamaschi E., Panizzolo M., Fenoglio I., Barbero F., Peters R., Undas A., Purker S., Giese B., Lalyer C.R. (2023). Nano- and microplastics: A comprehensive review on their exposure routes, translocation, and fate in humans. NanoImpact.

[25] Stiefel C., Stintzing F. (2023). Endocrine-active and endocrine-disrupting compounds in food—Occurrence, formation and relevance. NFS J..

[26] La Merrill M.A., Vandenberg L.N., Smith M.T., Goodson W., Browne P., Patisaul H.B., Guyton K.Z., Kortenkamp A., Cogliano V.J., Woodruff T.J. (2020). Consensus on the key characteristics of endocrine-disrupting chemicals as a basis for hazard identification. Nat. Rev. Endocrinol..

[27] Hampl R., Stárka L. (2020). Endocrine disruptors and gut microbiome interactions. Physiol. Res..

[28] Banushi B., Collova J., Milroy H. (2025). Epigenetic Echoes: Bridging Nature, Nurture, and Healing Across Generations. Int. J. Mol. Sci..

[29] Al Aboud N.M., Tupper C., Jialal I. (2025). Genetics, Epigenetic Mechanism. StatPearls.

[30] Sarsenova M., Kim Y., Raziyeva K., Kazybay B., Ogay V., Saparov A. (2022). Recent advances to enhance the immunomodulatory potential of mesenchymal stem cells. Front. Immunol..

[31] Rio P., Caldarelli M., Chiantore M., Ocarino F., Candelli M., Gasbarrini A., Gambassi G., Cianci R. (2024). Immune Cells, Gut Microbiota, and Vaccines: A Gender Perspective. Cells.

[32] Singh D.D. (2024). Epigenetic Mechanisms of Endocrine-Disrupting Chemicals in Breast Cancer and Their Impact on Dietary Intake. J. XenoBiot..

[33] Dubé-Zinatelli E., Cappelletti L., Ismail N. (2024). Vaginal Microbiome: Environmental, Biological, and Racial Influences on Gynecological Health Across the Lifespan. Am. J. Reprod. Immunol..

[34] Elkafas H., Walls M., Al-Hendy A., Ismail N. (2022). Gut and genital tract microbiomes: Dysbiosis and link to gynecological disorders. Front. Cell Infect. Microbiol..

[35] Gholiof M., Adamson-De Luca E., Wessels J.M. (2022). The female reproductive tract microbiotas, inflammation, and gynecological conditions. Front. Reprod. Health.

[36] Dabee S., Passmore J.A.S., Heffron R., Jaspan H.B. (2021). The Complex Link between the Female Genital Microbiota, Genital Infections, and Inflammation. Infect. Immun..

[37] Chee W.J.Y., Chew S.Y., Than L.T.L. (2020). Vaginal microbiota and the potential of Lactobacillus derivatives in maintaining vaginal health. Microb. Cell Fact..

[38] Armstrong E., Liu R., Pollock J., Huibner S., Udayakumar S., Irungu E., Ngurukiri P., Muthoga P., Adhiambo W., Yegorov S. (2025). Quantitative profiling of the vaginal microbiota improves resolution of the microbiota-immune axis. Microbiome.

[39] Zhu B., Spaine K.M., Edupuganti L., Matveyev A., Serrano M.G., Buck G.A. (2023). Characteristics of Vaginal Microbes and Classification of the Vaginal Microbiome. https://biorxiv.org/lookup/doi/10.1101/2023.08.16.553525.

[40] Dong W., Wang S., Wang X., Xu G., Liu Q., Li Z., Lv N., Pan Y., Xiong Q., Liu D. (2024). Characteristics of Vaginal Microbiota of Women of Reproductive Age with Infections. Microorganisms.

[41] Dong M., Dong Y., Bai J., Li H., Ma X., Li B., Wang C., Li H., Qi W., Wang Y. (2023). Interactions between microbiota and cervical epithelial, immune, and mucus barrier. Front. Cell Infect. Microbiol..

[42] Valeriano V.D., Lahtinen E., Hwang I.-C., Zhang Y., Du J., Schuppe-Koistinen I. (2024). Vaginal dysbiosis and the potential of vaginal microbiome-directed therapeutics. Front. Microbiomes.

[43] Holdcroft A.M., Ireland D.J., Payne M.S. (2023). The Vaginal Microbiome in Health and Disease-What Role Do Common Intimate Hygiene Practices Play?. Microorganisms.

[44] Gliniewicz K., Schneider G.M., Ridenhour B.J., Williams C.J., Song Y., Farage M.A., Miller K., Forney L.J. (2019). Comparison of the Vaginal Microbiomes of Premenopausal and Postmenopausal Women. Front. Microbiol..

[45] Brotman R.M., Shardell M.D., Gajer P., Fadrosh D., Chang K., Silver M.I., Viscidi R.P., Burke A.E., Ravel J., Gravitt P.E. (2014). Association between the vaginal microbiota, menopause status, and signs of vulvovaginal atrophy. Menopause.

[46] Hillier S.L., Lau R.J. (1997). Vaginal Microflora in Postmenopausal Women Who Have Not Received Estrogen Replacement Therapy. Clin. Infect. Dis..

[47] Song S.D., Acharya K.D., Zhu J.E., Deveney C.M., Walther-Antonio M.R.S., Tetel M.J., Chia N. (2020). Daily Vaginal Microbiota Fluctuations Associated with Natural Hormonal Cycle, Contraceptives, Diet, and Exercise. mSphere.

[48] Yoo J.Y., Groer M., Dutra S.V.O., Sarkar A., McSkimming D.I. (2020). Gut Microbiota and Immune System Interactions. Microorganisms.

[49] Mendz G.L. (2023). The Vaginal Microbiome during Pregnancy in Health and Disease. Appl. Microbiol..

[50] Gao H., Liu Q., Wang X., Li T., Li H., Li G., Tan L., Chen Y. (2024). Deciphering the role of female reproductive tract microbiome in reproductive health: A review. Front. Cell Infect. Microbiol..

[51] Baker J.M., Al-Nakkash L., Herbst-Kralovetz M.M. (2017). Estrogen–gut microbiome axis: Physiological and clinical implications. Maturitas.

[52] Shanle E.K., Xu W. (2011). Endocrine disrupting chemicals targeting estrogen receptor signaling: Identification and mechanisms of action. Chem. Res. Toxicol..

[53] Wang H., Shi F., Zheng L., Zhou W., Mi B., Wu S., Feng X. (2025). Gut microbiota has the potential to improve health of menopausal women by regulating estrogen. Front. Endocrinol..

[54] Jandhyala S.M., Talukdar R., Subramanyam C., Vuyyuru H., Sasikala M., Nageshwar Reddy D. (2015). Role of the normal gut microbiota. World J. Gastroenterol..

[55] Wiertsema S.P., van Bergenhenegouwen J., Garssen J., Knippels L.M.J. (2021). The Interplay between the Gut Microbiome and the Immune System in the Context of Infectious Diseases throughout Life and the Role of Nutrition in Optimizing Treatment Strategies. Nutrients.

[56] Bhol N.K., Bhanjadeo M.M., Singh A.K., Dash U.C., Ojha R.R., Majhi S., Duttaroy A.K., Jena A.B. (2024). The interplay between cytokines, inflammation, and antioxidants: Mechanistic insights and therapeutic potentials of various antioxidants and anti-cytokine compounds. Biomed. Pharmacother..

[57] Zhang H., Yang Lee B.J., Wang T., Xiang X., Tan Y., Han Y., Bi Y., Zhi F., Wang X., He F. (2025). Microbiota, chronic inflammation, and health: The promise of inflammatome and inflammatomics for precision medicine and health care. hLife.

[58] Shen Y., Fan N., Ma S.X., Cheng X., Yang X., Wang G. (2025). Gut Microbiota Dysbiosis: Pathogenesis, Diseases, Prevention, and Therapy. MedComm.

[59] Meir M., Burkard N., Ungewiß H., Diefenbacher M., Flemming S., Kannapin F., Germer C.-T., Schweinlin M., Metzger M., Waschke J. (2019). Neurotrophic factor GDNF regulates intestinal barrier function in inflammatory bowel disease. J. Clin. Invest..

[60] Sarkar A., McInroy C.J.A., Harty S., Raulo A., Ibata N.G.O., Valles-Colomer M., Johnson K.V.-A., Brito I.L., Henrich J., Archie E.A. (2024). Microbial transmission in the social microbiome and host health and disease. Cell.

[61] Metwaly A., Kriaa A., Hassani Z., Carraturo F., Druart C., Arnauts K., Wilmes P., Walter J., Consortium S.R., IHMCSA Consortium (2025). A Consensus Statement on establishing causality, therapeutic applications and the use of preclinical models in microbiome research. Nat. Rev. Gastroenterol. Hepatol..

[62] Wu L., Zhang J., Xin Y., Ma J., Chen T., Nie J., Niu P. (2024). Associations between phenols, parabens, and phthalates and depressive symptoms: The role of inflammatory markers and bioinformatic insights. Ecotoxicol. Environ. Saf..

[63] Calero-Medina L., Jimenez-Casquet M.J., Heras-Gonzalez L., Conde-Pipo J., Lopez-Moro A., Olea-Serrano F., Mariscal-Arcas M. (2023). Dietary exposure to endocrine disruptors in gut microbiota: A systematic review. Sci. Total Environ..

[64] Calvigioni M., Mazzantini D., Celandroni F., Ghelardi E. (2023). Animal and In Vitro Models as Powerful Tools to Decipher the Effects of Enteric Pathogens on the Human Gut Microbiota. Microorganisms.

[65] Cheng Q., Chen S. (2025). Using In Vitro Models to Study the Interactions Between Environmental Exposures and Human Microbiota. Microorganisms.

[66] Ayehunie S., Islam A., Cannon C., Landry T., Pudney J., Klausner M., Anderson D.J. (2015). Characterization of a Hormone-Responsive Organotypic Human Vaginal Tissue Model: Morphologic and Immunologic Effects. Reprod. Sci. Thousand Oaks. Calif..

[67] Castellanos-Ruiz D., Ojeda-Borbolla J.G., Ruiz-García O.V., Peña-Corona S.I., Martínez-Peña A.A., Ibarra-Rubio M.E., Mendoza-Rodríguez A. (2025). Uterine Microbiota and Bisphenols: Novel Influencers in Reproductive Health. J. Xenobiot..

[68] O’Mahony S.M., Comizzoli P. (2023). Special series on the role of the microbiome in reproduction and fertility. Reprod. Fertil..

[69] Kleine Bardenhorst S., Berger T., Klawonn F., Vital M., Karch A., Rübsamen N. (2021). Data Analysis Strategies for Microbiome Studies in Human Populations—A Systematic Review of Current Practice. mSystems.

[70] Ahmad A., Imran M., Ahsan H. (2023). Biomarkers as Biomedical Bioindicators: Approaches and Techniques for the Detection, Analysis, and Validation of Novel Biomarkers of Diseases. Pharmaceutics.

[71] Cordelli E., Ardoino L., Benassi B., Consales C., Eleuteri P., Marino C., Sciortino M., Villani P., Brinkworth M.H., Chen G. (2024). Effects of radiofrequency electromagnetic field (RF-EMF) exposure on male fertility: A systematic review of experimental studies on non-human mammals and human sperm in vitro. Environ. Int..

[72] Stahl G.K., Maznevski M.L. (2021). Unraveling the effects of cultural diversity in teams: A retrospective of research on multicultural work groups and an agenda for future research. J. Int. Bus. Stud..

[73] Yang S.Y., Han S.M., Lee J.Y., Kim K.S., Lee J.E., Lee D.W. (2025). Advancing Gut Microbiome Research: The Shift from Metagenomics to Multi-Omics and Future Perspectives. J. Microbiol. Biotechnol..

[74] Sonnenburg E.D., Smits S.A., Tikhonov M., Higginbottom S.K., Wingreen N.S., Sonnenburg J.L. (2016). Diet-induced extinctions in the gut microbiota compound over generations. Nature.

[75] Punzón-Jiménez P., Labarta E. (2021). The impact of the female genital tract microbiome in women health and reproduction: A review. J. Assist. Reprod. Genet..

[76] Abou Chacra L., Fenollar F., Diop K. (2021). Bacterial Vaginosis: What Do We Currently Know?. Front. Cell Infect. Microbiol..

[77] Kairys N., Carlson K., Garg M. (2025). Bacterial Vaginosis. StatPearls.

[78] Balakrishnan S.N., Yamang H., Lorenz M.C., Chew S.Y., Than L.T.L. (2022). Role of Vaginal Mucosa, Host Immunity and Microbiota in Vulvovaginal Candidiasis. Pathogens.

[79] Saadaoui M., Singh P., Ortashi O., Al Khodor S. (2023). Role of the vaginal microbiome in miscarriage: Exploring the relationship. Front. Cell Infect. Microbiol..

[80] Balla B., Illés A., Tobiás B., Pikó H., Beke A., Sipos M., Lakatos P., Kósa J.P. (2024). The Role of the Vaginal and Endometrial Microbiomes in Infertility and Their Impact on Pregnancy Outcomes in Light of Recent Literature. Int. J. Mol. Sci..

[81] López-Moreno A., Aguilera M. (2021). Vaginal Probiotics for Reproductive Health and Related Dysbiosis: Systematic Review and Meta-Analysis. J. Clin. Med..

[82] Zlatnik M.G. (2016). Endocrine-Disrupting Chemicals and Reproductive Health. J. Midwifery Womens Health.

[83] Goin D.E., Abrahamsson D., Wang M., Jiang T., Park J.-S., Sirota M., Morello-Frosch R., DeMicco E., Zlatnik M.G., Woodruff T.J. (2022). Disparities in chemical exposures among pregnant women and neonates by socioeconomic and demographic characteristics: A nontargeted approach. Environ. Res..

[84] Escorcia Mora P., Valbuena D., Diez-Juan A. (2025). The Role of the Gut Microbiota in Female Reproductive and Gynecological Health: Insights into Endometrial Signaling Pathways. Life.

[85] Baud A., Hillion K.-H., Plainvert C., Tessier V., Tazi A., Mandelbrot L., Poyart C., Kennedy S.P. (2023). Microbial diversity in the vaginal microbiota and its link to pregnancy outcomes. Sci. Rep..

[86] Sethi N., Narayanan V., Saaid R., Ahmad Adlan A.S., Ngoi S.T., Teh C.S.J., Hamidi M. (2025). Prevalence, risk factors, and adverse outcomes of bacterial vaginosis among pregnant women: A systematic review. BMC Pregnancy Childbirth.

[87] Obuobi S., Škalko-Basnet N. (2024). Understanding vaginal biofilms: The first step in harnessing antimicrobial nanomedicine. J. Control. Release.

[88] Oliveira M., Antunes W., Mota S., Madureira-Carvalho Á., Dinis-Oliveira R.J., Dias Da Silva D. (2024). An Overview of the Recent Advances in Antimicrobial Resistance. Microorganisms.

[89] Li Y., Chen L., Zhou N., Chen Y., Ling Z., Xiang P. (2024). Microplastics in the human body: A comprehensive review of exposure, distribution, migration mechanisms, and toxicity. Sci. Total Environ..

[90] Srivastava U., Kesheri M., Kanchan S., Singh S., Kesheri M., Kanchan S., Salisbury T.B., Sinha R.P. (2024). Computational Omics Protocol for the Comparative Study of Microbiome Analysis. Microbial Omics in Environment and Health.

[91] Mundinger C., Schulz N.K.E., Singh P., Janz S., Schurig M., Seidemann J., Kurtz J., Müller C., Schielzeth H., Von Kortzfleisch V.T. (2025). Testing the reproducibility of ecological studies on insect behavior in a multi-laboratory setting identifies opportunities for improving experimental rigor. Fetter-Pruneda IA, editor. PLoS Biol..

[92] O’Callaghan K.M., Roth D.E. (2020). Standardization of laboratory practices and reporting of biomarker data in clinical nutrition research. Am. J. Clin. Nutr..

[93] Pantazi A.C., Balasa A.L., Mihai C.M., Chisnoiu T., Lupu V.V., Kassim M.A.K., Mihai L., Frecus C.E., Chirila S.I., Lupu A. (2023). Development of Gut Microbiota in the First 1000 Days after Birth and Potential Interventions. Nutrients.

[94] Kennedy M.S., Chang E.B. (2020). The microbiome: Composition and locations. Prog. Mol. Biol. Transl. Sci..

[95] Campana A.M., Laue H.E., Shen Y., Shrubsole M.J., Baccarelli A.A. (2022). Assessing the role of the gut microbiome at the interface between environmental chemical exposures and human health: Current knowledge and challenges. Environ. Pollut..

[96] Coppola F., Fratianni F., Bianco V., Wang Z., Pellegrini M., Coppola R., Nazzaro F. (2025). New Methodologies as Opportunities in the Study of Bacterial Biofilms, Including Food-Related Applications. Microorganisms.

[97] Baccarelli A., Dolinoy D.C., Walker C.L. (2023). A precision environmental health approach to prevention of human disease. Nat. Commun..

[98] Dong Y.H., Luo Y.H., Liu C.J., Huang W.Y., Feng L., Zou X.Y., Zhou J.-Y., Li X.-R. (2024). Changes in microbial composition and interaction patterns of female urogenital tract and rectum in response to HPV infection. J. Transl. Med..

[99] Abdool Karim S.S., Baxter C., Passmore J.A.S., McKinnon L.R., Williams B.L. (2019). The genital tract and rectal microbiomes: Their role in HIV susceptibility and prevention in women. J. Int. AIDS Soc..

